# FGF signaling controls caudal hindbrain specification through Ras-ERK1/2 pathway

**DOI:** 10.1186/1471-213X-9-61

**Published:** 2009-12-03

**Authors:** Ferran Aragon, Cristina Pujades

**Affiliations:** 1 Departament de Ciències Experimentals i de la Salut, Universitat Pompeu Fabra, Parc de Recerca Biomèdica de Barcelona, PRBB, Barcelona, Spain

## Abstract

**Background:**

During early steps of embryonic development the hindbrain undergoes a regionalization process along the anterior-posterior (AP) axis that leads to a metameric organization in a series of rhombomeres (r). Refinement of the AP identities within the hindbrain requires the establishment of local signaling centers, which emit signals that pattern territories in their vicinity. Previous results demonstrated that the transcription factor *vHnf1 *confers caudal identity to the hindbrain inducing *Krox20 *in r5 and *MafB/Kreisler *in r5 and r6, through FGF signaling [1].

**Results:**

We show that in the chick hindbrain, *Fgf3 *is transcriptionally activated as early as 30 min after *mvHnf1 *electroporation, suggesting that it is a direct target of this transcription factor. We also analyzed the expression profiles of FGF activity readouts, such as *MKP3 *and *Pea3*, and showed that both are expressed within the hindbrain at early stages of embryonic development. In addition, *MKP3 *is induced upon overexpression of *mFgf3 *or *mvHnf1 *in the hindbrain, confirming *vHnf1 *is upstream FGF signaling. Finally, we addressed the question of which of the FGF-responding intracellular pathways were active and involved in the regulation of *Krox20 *and *MafB *in the hindbrain. While Ras-ERK1/2 activity is necessary for *MKP3, Krox20 and MafB *induction, PI3K-Akt is not involved in that process.

**Conclusion:**

Based on these observations we propose that *vHnf1 *acts directly through FGF3, and promotes caudal hindbrain identity by activating *MafB *and *Krox20 *via the Ras-ERK1/2 intracellular pathway.

## Background

The hindbrain is the most posterior vesicle of the embryonic brain. During early steps of neural development, the hindbrain is transiently organized in segments along the anterior-posterior (AP) axis, which are called rhombomeres (r). This transient segmental organization is necessary for the correct specification of the different neuronal subtypes, the location of the cranial nerve exit points, and the migration streams of the neural crest cells from the dorsal hindbrain towards the branchial arches. Rhombomeres display a specific combinatory of gene expression that confers molecular identity to the rhombomeric territories, and they are compartment-like units with cell lineage restriction (for reviews see [2,3]).

Refinement of the AP identities within the hindbrain requires the establishment of local signaling centers, which emit signals that pattern territories in their vicinity. Two signaling centers which emit FGF and WNT signals are located within the hindbrain: the Isthmic Organizer (IsO), at the level of the Midbrain-Hindbrain Boundary (MHB) (for review see [3,4]), and the 'r4-FGF source' [5]. FGFs emitted from the central and caudal hindbrain have been demonstrated to be crucial for hindbrain specification. In zebrafish, *fgf3 *and *fgf8 *from r4 have redundant functions in patterning the hindbrain [6,7], whereas in chick and mouse *Fgf3 *dynamically expressed in the r4-r6 region is needed for the specification of the caudal hindbrain [1,8-10]. Gain-of-function experiments in zebrafish suggested that FGFs from the hindbrain cooperate with the transcription factor *vHnf1 *in the specification of the caudal hindbrain [11,12]. This cooperation occurs early during neurulation and leads to the induction of two genes involved in rhombomeric specification, *Krox20 *for r5 and *MafB *for r5 and r6. Results in chick suggest that *vHnf1 *operates upstream of FGF signaling in this regulation: *vHnf1 *not only cooperates with *Fgf3 *in the induction of *Krox20 *and *MafB *, but also regulates *Fgf3 *expression [1]. Analyses of the *Krox20 *and *MafB *regulatory regions in mice have shown that they contain functional vHNF1-binding sites, suggesting that *vHnf1 *can control these genes in a direct manner as well [13,14].

One of the questions that have challenged developmental biologists in the last years is how FGF signaling can generate such a different array of responses in the several developmental events in which is involved. It is known that these very diverse outcomes are context dependent, with FGF signaling acting in a cellular environment defined by previous and current signaling activities [15]. One of the most accepted hypotheses considers that the activation and tuning of different intracellular pathways downstream FGF signaling can generate part of this variability. Among those, the FGF-downstream intracellular cascades Ras-ERK1/2 and PI3K-Akt are those that have mostly been related to embryonic patterning events. Different and in some cases contradictory models have been proposed for the involvement of Ras-ERK1/2 and PI3K-Akt pathways in different tissues and systems [16-20]. In addition, the FGF signaling system is tightly regulated by a series of modulators, which exert their functions at different levels of the pathway, from the FGFR to specific components of the different intracellular pathways (reviewed in [21]). The expression of these genes is induced by FGF activity itself and regional and temporal variation in their levels of expression is though to tune FGF signaling to the appropriate levels for each particular event. The term 'synexpression group' has been adopted to designate sets of genes that share complex spatio-temporal expression patterns and have a functional relationship [22]. Synexpression groups form expression cassettes that can be found at different times and locations during development. The FGF factors, such as *Fgf8 *and *Fgf4*, and FGFRs (*FGFR1-4*), together with negative modulators of FGF signaling (*MKP3, SPRY2*, *Sef *and *Spred *), the positive modulator *FLRT3 *and transcription factors such as the members of the Ets-type family *Pea3, Erm *and *Er81 *have been designated as the 'FGF synexpression group' [21,23]. The aim of the present work was to dissect the FGF-intracellular cascade involved in caudal hindbrain patterning. Further to our previous observations on the regulation of *Fgf3 *in the chick hindbrain, we provide evidence suggesting that *Fgf3 *is a direct transcriptional target of *vHnf1*. We provide a detailed spatial and temporal map of expression of key components of the FGF signaling system such as *MKP3*, *Pea3 *and of Ras-ERK1/2 and PI3K-Akt activity in the hindbrain at early stages of embryonic development. We also analyze the functional activity of the intracellular pathways downstream FGF signaling using specific chemical inhibitors. The results show that *MKP3 *expression colocalizes with activated ERK1/2 and it is sensitive to inhibition of Ras-ERK1/2. This pathway mediates the functions of FGF3 signaling in caudal hindbrain specification regulating the expression of *Krox20 *and *MafB *, without involvement of the PI3K-Akt pathway. Altogether these data provide new insights in the repertoire of FGF pathway genes associated to hindbrain development and on the role of FGF signaling in caudal hindbrain specification.

## Results

### Fgf3 is rapidly induced after vHnf1 overexpression

**Figure 1:**
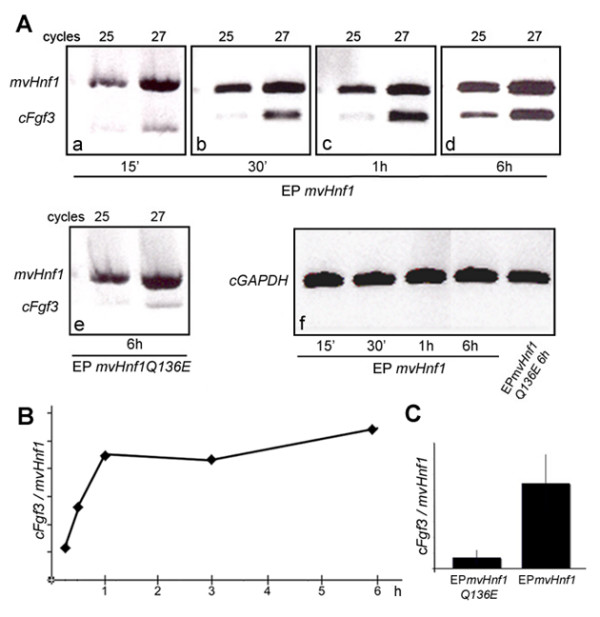
***Fgf3 *is rapidly induced after *mvHnf1 *overexpression**. Embryos were electroporated with *mvHnf1-GFP *construct and incubated during different time periods. After the desired incubation period the hindbrain tissue was processed for total RNA extraction and one-step RT-PCR amplification for *cFgf3 *and *mvHnf1*. (A) Amplified bands corresponding to *cFgf3 *and *mvHnf1 *checked after 25 and 27 cycles. Samples correspond to 15 min (a), 30 min (b), 1 h (c) and 6 h (d) after *mvHnf1 *overexpression, or 6 h after *mvHnf1-Q136E *overexpression (e). Amplification of *GAPDH *was run in parallel to normalize sample values (f). (B) The ratio of intensity between the *cFgf3 *and the *mvHnf1 *band at each time point was calculated and plotted in the graphic, in order to analyze the relative level of *cFgf3 *expression. A quantification program was used to calculate the intensity of each band and it was expressed in percentage of volume of the band (*cFgf3/mvHnf1*, see M&M). (C) Comparison of *cFgf3/mvHnf1 *ratio after *mvHnf1 *or *mvHnf1-Q136E *overexpression.

Previous work indicated that *Fgf3 *was downstream of *vHnf1 *in the induction of caudal rhombomeric markers such as *Krox20 *and *MafB *. *vHnf1 *operates in a specific time-window and is not able to induce *Fgf3*, *MafB *or *Krox20 *at late stages of hindbrain patterning [1]. One important question was to know whether *Fgf3 *was a direct downstream target of *vHnf1*. To address this issue we studied the time course of *Fgf3 *induction after *mvHnf1 *overexpression. We designed a semiquantitative RT-PCR approach to determine the kinetics of *Fgf3 *induction. Embryos of 3-4ss (HH8) were electroporated with *mvHnf1 *and incubated at 38°C during different time periods (15 min, 30 min, 1 h, 3 h, 6 h). After incubation, the hindbrain tissue was isolated and processed for RT-PCR amplification. 15 min after electroporation, a band of 600 bp corresponding to *mvHnf1 *expression at both 25 and 27 cycles was amplified (Fig. 1Aa, B), indicating the amount of ectopic *mvHnf1 *already transcribed in the embryo. The number of cycles used, 25 and 27, was to show that we were not under saturation conditions. At the same time point, a weaker 450 bp band corresponding to the endogenous *cFgf3 *expression was amplified by RT-PCR (Fig. 1Aa, B). To avoid biased results and to be able to compare between different time points, the ratio of intensity *cFgf3/mvHnf1 *was always used to analyze the data. As incubation time increased (30 min and 1 h), the intensity of the *cFgf3 *band was progressively stronger in respect to the *mvHnf1 *band (Fig. 1Ab-c, B). From 1 h onwards, the intensity of the *cFgf3 *band was relatively similar for all time points, suggesting that either the reaction reached saturation or *vHnf1 *was not able to induce *cFgf3 *transcription anymore (Fig. 1Ad, B). Thus, as early as 30 min after electroporation the endogenous expression of *Fgf3 *was increased and by 1 h a plateau was reached (Fig. 1B). Control experiments were performed with a form of *vHnf1 *containing the Q136E substitution in the POU-specific domain that completely abolishes DNA-binding [1,24]. The construct was overexpressed and embryos were incubated at 38°C during 6 h (Fig. 1Ae). The *cFgf3 *relative levels of expression were much higher in experimental samples than in the control (compare Fig. 1Ad and 1Ae, Fig. 1C for quantification).

These results suggest that *vHnf1 *positively regulates *Fgf3 *in the chick hindbrain. The RT-PCR semiquantitative analysis shows that *Fgf3 *is rapidly induced after *vHnf1 *overexpression, suggesting a direct transcriptional regulation. Although regulatory analysis of the mouse *Fgf3 *gene revealed a DNA region driving *Fgf3 *expression to the hindbrain, this enhancer region does not fully recapitulate the dynamics of *Fgf3 *expression within the hindbrain and it did not bring much light into the transcription factor binding candidates [25].

### Akt and ERK1/2 pathways are active in the caudal hindbrain

The FGF signals exert their function by activating different intracellular pathways. Although five intracellular pathways are known to be downstream of FGF signaling, mainly Ras-ERK1/2 and, to a lesser extent, PI3K-Akt have been related to embryonic processes in different tissues and models [16-19]. Given that FGF signaling controls *Krox20 *and *MafB *expression [1,10], our next question was to study which of these intracellular networks were activated downstream *Fgf3 *in the caudal hindbrain.

**Figure 2:**
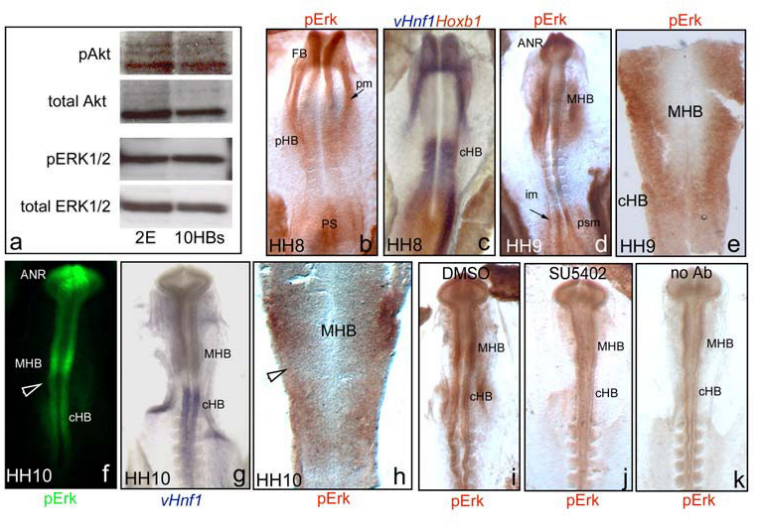
**Phosphorylated forms of Akt and ERK1/2 are present in the caudal hindbrain**. (a) Western Blot analysis of protein extracts of whole embryos (left column) and isolated caudal hindbrain (right column). Phosphorylated (activated) forms of both Akt and ERK1/2 were present in protein extracts from the caudal hindbrain. Total forms of Akt and ERK1/2 were used as loading controls. pERK1/2 immunodetection is shown in red (b, d-e, h-k) or in green fluorescence in (f). *In situ *hybridization with *vHnf1-Hoxb1 *(c) and *vHnf1 *(g) to position the caudal hindbrain. (i-k) Control experiments to show the specificity of pErk staining: embryos incubated with DMSO (i) or with SU5402 (j), and assayed for pErk (note that specific pErk staining in the MHB and in the caudal hindbrain disappears upon SU5402 treatment); (k) embryos treated as in (i) but without pErk-Ab incubation, as negative control. Whole-mount embryos (b-d, f-g, i-k), or flat-mounted hindbrains (e, h) at indicated stages. ANR, anterior neural ridge; cHB, caudal hindbrain; FB, forebrain; HB, hindbrain; im, intermediate mesoderm; MHB, midbrain-hindbrain boundary; pm, precardiac mesoderm; ps: primitive strike; psm, presomitic mesoderm. Anterior is at the top.

For this purpose, the activated forms of FGF effectors within the hindbrain at early stages of embryonic development were analyzed. Caudal hindbrain tissue from HH8-HH9 stage embryos was isolated and western blot analysis was performed to detect the Akt and ERK1/2 phosphorylated forms (pAkt and pERK1/2). When antibodies against both the total and the phosphorylated forms of Akt were used in protein extracts of isolated hindbrains, a 60 kD band corresponding to Akt was obtained in both cases (Fig. 2a). This result suggests that the PI3K-Akt pathway is active at these stages of hindbrain patterning. Western blot analysis detecting either the total or the phosphorylated forms of ERK1/2 revealed that Ras-ERK1/2 pathway was also active during this period (Fig. 2a). As positive controls for ERK1/2 and Akt activation, protein extracts of whole embryos were used (Fig. 2a).

**Figure 3:**
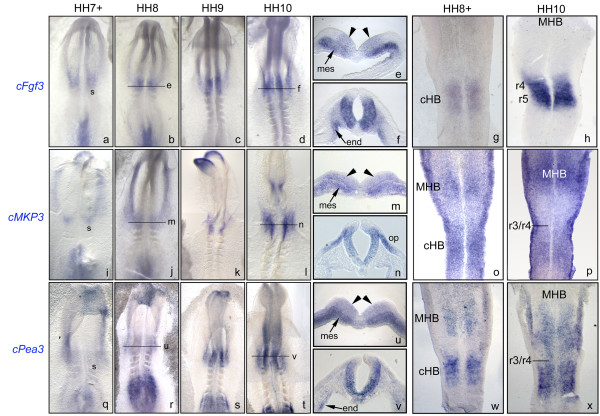
**Genes of the FGF synexpression group are expressed in the caudal hindbrain**. *In situ *hybridization of embryos between HH7^+ ^and HH10 with: *cFgf3 *(a-h), *cMKP3 *(i-p), and *cPea3 *(q-x). Arrowheads in (e), (m) and (u) point to early expression in the neural plate. Pictures show whole-mount embryos (a-d, i-l,q-t), flat-mounted hindbrains (g, h,o,p,w,x) and transverse sections (e, f,m,n,u,v) whose correspondences to whole-mounts are specified in the pictures. cHB, caudal hindbrain; end, endoderm; mes, mesoderm; MHB, midbrain hindbrain boundary; op, otic placode; r, rhombomere; s, somite. Anterior is at the top.

Next, we wanted to analyze the spatial distribution of the FGF activities. With this in mind, we performed whole-mount immunodetection of pERK1/2 with embryos at HH8-HH10 stages. As shown in Fig. 2b-h, pERK1/2 was detected in embryonic territories within or close to FGF reported sources such as: the anterior neural ridge (ANR), the mid-hindbrain boundary (MHB), the precardiac mesoderm (pm), the primitive strike (ps), the caudal neural plate, and the presomitic and intermediate mesoderm (Fig. 2b-e; [20]). Between HH8 and HH10 *Fgf3 *is expressed in a domain corresponding to r4 and r5 (Fig. 3; [1,26]). Coincident with this expression domain, the Ras-ERK1/2 pathway was also active in the hindbrain at 4ss (HH8) (Fig. 2b). By HH9 pERK1/2 persisted in the caudal hindbrain and it was extended to the rostral hindbrain, the MHB and the caudal midbrain (Fig. 2b, d). Flat-mounted hindbrains in Fig. 2e showed that pERK1/2 was excluded from the ventral part of the tube, with the exception of the caudal hindbrain, where it was localized all along the dorsoventral axis. At HH10, Ras-ERK1/2 activity was still maintained in the hindbrain (Fig. 2f-h). The strongest activity was localized in the MHB (Fig. 2f-h). Unfortunately, it was not possible to analyze the pAkt spatial distribution since available antibodies did not work in whole-mount immunostainings.

### Expression of the readouts of FGF activity in the caudal hindbrain

To further investigate the FGF signaling system during hindbrain patterning, we checked for the expression of genes of the synexpression group that could act as readouts of FGF activity, to study whether they recapitulate the *Fgf3 *expression profile and, therefore, can be used as local readouts of the FGF3 activity in the caudal hindbrain.

*Fgf3 *is the most strongly expressed *Fgf *during hindbrain patterning displaying a dynamic pattern of expression [26]. At 2ss (HH7+), *Fgf3 *transcripts were already present in the prospective hindbrain (Fig. 3a). At HH8 and HH9 *Fgf3 *expression was maintained in the caudal hindbrain occupying a domain corresponding to the presumptive r4-r5 territory (Fig. 3b, g). Transverse sections show that at this stage, *Fgf3 *expression was restricted to the ventral part of the hindbrain (Fig. 3e, arrowheads), with the exception of the floor plate, and to the hindbrain-underlying mesoderm (Fig. 3e, see arrow). At HH10 *Fgf3 *was still expressed in r4 and r5 (Fig. 3d, h) and in the endoderm that gives rise to the pharyngeal pouches (Fig. 3d, f, arrow).

*MKP3 *(MAPK Phosphatase 3) is a phosphatase belonging to the Ras-ERK1/2 pathway that is commonly regulated by FGF signaling [16,17,27,28]. *MKP3 *works as negative modulator of the MAPK pathway by specifically dephosphorylating ERK1/2 [29,30]. At 2ss (HH7+), *MKP3 *was faintly detected in the prospective hindbrain (Fig. 3i). At HH8 and HH9, it was apparent in the ANR and maintained in the caudal hindbrain (Fig. 3j, o). The *MKP3 *expression domain was broader than the *Fgf3*-expressing area (compare flat-mounts in Fig. 3g, o). *MKP3 *transcripts were detected in the presumptive MHB territory at HH10 (Fig. 3l, p), where *Fgf8 *is expressed [31]. Transverse sections confirmed that *MKP3 *was indeed expressed in the neural plate (Fig. 3m, arrowheads), and in the mesoderm underlying the hindbrain (Fig. 3m, arrows), where *Fgf3 *and *Fgf19 *are known to be expressed (Fig. 3e; [32]). *MKP3 *expression was maintained in the hindbrain up to r3/r4 (Fig. 3l, p). *MKP3 *was expressed along the DV axis of the neural tube (Fig. 3p), and it was downregulated in the underlying mesoderm by HH10 (Fig. 3n). The ectoderm corresponding to the otic placode was also positive for *MKP3 *expression (Fig. 3n).

*Pea3 *(*Polyoma enhancer activator 3*)is a transcription factor belonging to the Pea3 subfamily of the Ets transcription factors. It is a common mediator of the FGF activity into the nucleus and its expression is also regulated by FGF signaling [33]. *Pea3 *was weakly detected in the prospective hindbrain by 2ss (HH7+) (Fig. 3q). At HH8 and HH9, *Pea3 *expression increased in the caudal hindbrain (Fig. 3r-s, w). Although at HH7+ *Pea3 *was very faintly expressed in the neural plate and strongly in the mesoderm (Fig. 3q, u), by HH10 *Pea3 *expression was mainly confined to the hindbrain and the midbrain (Fig. 3t, x), with no expression in the underlying mesoderm (Fig. 3v). Note that contrary to *MKP3*, *Pea3 *was not expressed in the otic ectoderm (Fig. 3v).

*Sprouty2 *(*Spry2*), as *MKP3*, is a negative modulator of the MAPK pathway regulated by FGF signaling. It is known to be expressed by HH10 in the MHB and r1 [34]. When the expression of *Sprouty2 *was analyzed within the hindbrain, two patches of *Sprouty2 *expression were observed. The most anterior one corresponded to the MHB and rostral hindbrain, whilst the most posterior patch, that was very faint, was in a domain of the hindbrain not fully coincident with pERK1/2 activation or *Fgf3 *expression (data not shown).

These results indicate that genes of the FGF synexpression group are expressed in the caudal hindbrain. At early stages of hindbrain patterning, HH7-HH9, *MKP3 *and *Pea3 *coincided with *Fgf3 *expression in the hindbrain. By HH10, *MKP3 *was still maintained in the hindbrain in an area coincident with *Fgf3 *expression; on the other hand, *Pea3 *was expanded in the hindbrain and in the midbrain. *SPRY2 *expression in the caudal hindbrain was very transient and not fully coincident with the *Fgf3*.

### MKP3 is a readout of FGF3 and vHnf1 functions in the hindbrain

**Figure 4:**
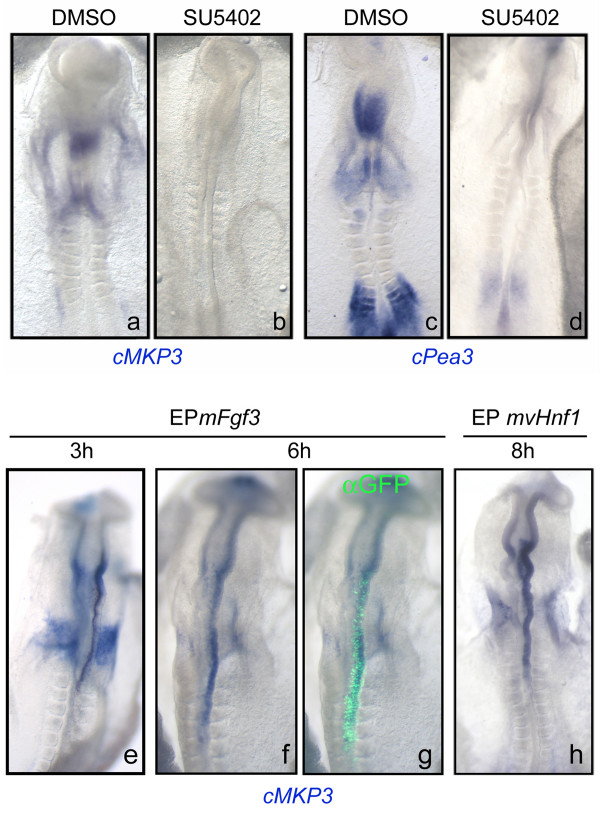
***Pea3 *and *MKP3 *expression are FGF-dependent**. Embryos were explanted and cultured during 2 h in control medium (a, c) or medium supplemented with 25 μM SU5402 (b, d) and assayed for expression of *cMKP3 *or *cPea3*. Embryos were electroporated with *mFgf3 *(e-g) or *mvHnf1 *(h), incubated during 3 h, 6 h or 8 h and then analyzed for *cMKP3 *(e-h) and for anti-GFP (g). Electroporated side is the right one. (g) Merge of *MKP3 *in situ hybridization and anti-GFP staining to show the extension of the electroporation and the overlapping of GFP and ectopic *MKP3*. All the pictures show whole-mount embryos with anterior to the top.

We have shown that at HH7-HH9 stages *MKP3 *and *Pea3 *expression in the caudal hindbrain coincided with *Fgf3 *expression and ERK1/2 activation. In order to confirm that these genes are indeed dependent on FGF signals within the hindbrain we analyzed their expression after blocking FGF signaling. As expected, when FGF signaling was blocked in HH9 embryos by incubation with medium containing 25 μM SU5402 during 2 h, both *MKP3 *and *Pea3 *expression were completely abolished (Fig. 4a-d, n = 5/5 for *MKP3 *and n = 2/2 for *Pea3*). This is in agreement with several studies that show that expression of both *MKP3 *and *Pea3 *is FGF-dependent [18,27,28,33,35-37].

Most of the reports suggested MKP3 as a readout of FGF8 activity. Since *Fgf8 *is not expressed in the caudal hindbrain at this stage, we wanted to address whether *Fgf3 *was sufficient to mediate *MKP3 *induction. For this purpose, we overexpressed *mFgf3 *in the neural tube of HH8-9 embryos and analyzed *cMKP3 *expression. As early as 3 h after electroporation the expression domain of *MKP3 *in the hindbrain was expanded (Fig. 4e, n = 5/6) and by 6 h after *mFgf3 *electroporation, *MKP3 *was ectopically expressed in the entire hindbrain (Fig. 4f-g, n = 6/6). In addition, *mvHnf1 *overexpression was also able to induce *MKP3 *expression, although this effect was detected later, only 8 h after electroporation (Fig. 4h, n = 8/8).

In summary, the expression of *MKP3 *and *Pea3 *in the hindbrain relays on FGF signaling, and *Fgf3 *is able to ectopically induce *MKP3 *in the neural tube as soon as 3 h after overexpression. *vHnf1 *also induces *MKP3 *but delayed with respect to *Fgf3*. This observation, together with previous data that showed that *vHnf1 *activates *Fgf3 *(Fig. 1, [1]), suggests that *MKP3 *is induced by *vHnf1 *through *Fgf3 *and confirms that *vHnf1 *is upstream of FGF signaling.

### FGF activity in the hindbrain is mediated by the Ras-ERK1/2 pathway

Next step was to dissect the FGF intracellular pathways involved in hindbrain patterning. Different models are proposed for the involvement of Ras-ERK1/2 and PI3K-Akt pathways in patterning events. Most of them involve uniquely the ERK1/2 pathway [16,27,38]. PI3K-Akt is usually proposed to crosstalk with Ras-ERK1/2 in either synergistic [39] or antagonistic manners [17,36]. To understand the role of these pathways in the caudal hindbrain we used a functional approach. ERK1/2 activity was blocked with the chemical inhibitor PD184352 and PI3K function with LY2944002 [28,40]. For general blockade of FGF signaling, SU5402 was used [41].

**Figure 5:**
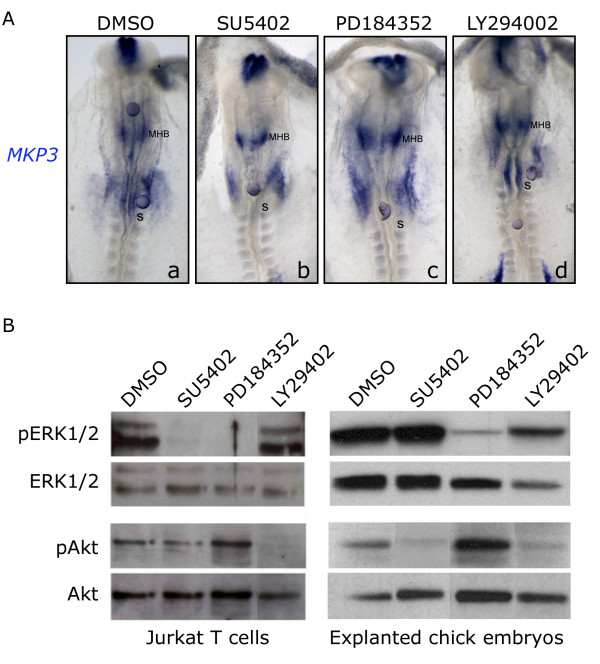
***MKP3 *is dependent on ERK1/2 but not Akt activation**. (A) Beads soaked with specific inhibitors were placed in the caudal hindbrain or in the presumptive MHB of HH7^+^-8 explanted embryos. Pharmacological treatments were as follows: DMSO (a), FGFR inhibitor SU5402 (b), ERK1/2 inhibitor PD184352 (c), PI3K inhibitor LY294002 (d). Explants were incubated during 6 h at 38°C. (B) Jurkat cells (left side lanes) and HH7-8 explanted embryos (right side lanes) were treated with DMSO, SU5402, PD184352 or LY294002 and analyzed by western blot for total and phosphorylated forms of Akt and ERK1/2. PD184352 treatment impeded ERK1/2 phosphorylation without affecting the PI3K-Akt pathway and, conversely, LY294002 treatment abolished Akt phosphorylation without affecting the Ras-ERK1/2 pathway. Embryos are shown in whole-mount with anterior to the top.

We analyzed how inhibition of ERK1/2 and PI3K pathways affected the hindbrain expression of *MKP3*, the readout of FGF activity. Formate beads were soaked in the specific inhibitors or in DMSO and then placed within the caudal hindbrain of HH7^+^-HH8 explanted embryos. Explants were incubated during 6 h at 38°C and then analyzed for *MKP3 *expression (Fig. 5A). When PD184252 coated beads were placed near the caudal hindbrain or the presumptive isthmus/MHB of HH8 embryos, *MKP3 *expression was completely abolished in these territories (Fig. 5Ac, n = 8/8). Conversely, *MKP3 *expression was unaffected when LY294002 (Fig. 5Ad, n = 4/4) or DMSO (Fig. 5Aa, n = 5/5) coated beads were implanted. These results suggest that in the caudal hindbrain, as in the early MHB, *MKP3 *is regulated by Ras-ERK1/2 but not by the PI3K-Akt pathway. MKP3 plays a crucial role in controlling Ras-ERK1/2 cascade by specifically dephosphorylating ERK1/2. Our results support the view that in the hindbrain *MKP3 *mediates a negative feedback in the Ras-ERK1/2 pathway [27,28].

To confirm the non-regulation of *MKP3 *by the Akt pathway in the hindbrain, we further validated the specificity and the efficiency of the pathway-specific inhibitors PD184352 and LY294002. For this purpose, we assayed their function in Jurkat T cells sensitive to be activated by both pathways [42-44]. Jurkat cells were treated and processed for protein extraction and western blot analysis was performed to reveal total and phosphorylated forms of Akt and ERK1/2. As expected, cells that were treated with PD184352 gave negative signal for pERK1/2 but not for pAkt. Conversely, cells that were treated with LY29402 did not phosphorylate Akt but did ERK1/2 (Fig. 5B). In all cases, total forms of ERK1/2 and Akt were detected (Fig. 5B). However, when Jurkat T cells were treated with SU5402, pErk1/2 was downregulated but pAkt levels were maintained (Fig. 5B). This is explained by the fact that Jurkat cells are PTEN mutants: PTEN is inactivated and as consequence, PI3K action in the activation of Akt is reinforced [45]. This can be reverted by the application of LY294002 which directly inhibits PI3K, but not by SU5402 which is more upstream in the pathway, just at the level of the RTKs (Receptor Tyrosin Kinases).

Next, we tested the inhibitors in embryonic explants. HH7^+^-HH8 embryos were explanted and incubated during 6 h at 38°C in the presence or absence of the inhibitors. They were processed for protein extraction and western blotted for phosphorylated and total forms of Akt and ERK1/2. Treatment with 20 μM PD184352 was able to specifically abolish ERK1/2 phosphorylation, while 40 μM LY294002 treatment abolished Akt phosphorylation (Fig. 5B). When explanted embryos were treated with SU5402, pAkt was downregulated but levels of pERK1/2 were maintained. This pERK1/2 maintenance was previously reported and attributed to a FGF-independent wounding response induced when the embryo is detached from the vitelline membrane during explantation [20,46]. These observations demonstrate that in the hindbrain the expression of the *MKP3 *is dependent on the Ras-ERK1/2 pathway, but independent of the PI3K-Akt activity.

**Figure 6:**
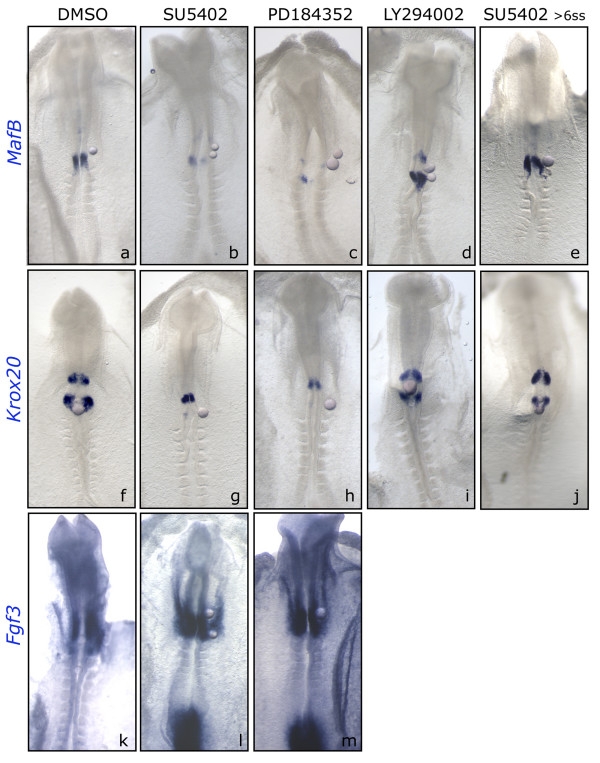
***MafB *and *Krox20 *are dependent on ERK1/2 but not on Akt activation**. HH7^+^-8 embryos (a-d, f-i,k-m) or HH9 (e, j) were explanted and beads soaked in specific inhibitors were placed in the caudal hindbrain. Afterwards, embryos were analyzed for *Krox20*, *MafB *or *Fgf3 *expression. Pharmacological treatments were as follows: DMSO (a, f,k), FGFR inhibitor SU5402 (b, e,g,j,l), ERK1/2 inhibitor PD184352 (c, h,m), or PI3K inhibitor LY294002 (d, i), during 6 h. Embryos are shown in whole-mount with anterior to the top.

Finally, our goal was to know which intracellular FGF-pathway was controlling hindbrain caudal identity. Loss-of-function experiments showed that *MafB *expression was inhibited when the ERK1/2 pathway was blocked with PD184352 (Fig. 6c, n = 8/8). Neither DMSO (Fig. 6a, n = 8/8) nor LY294002 (Fig. 6d, n = 5/5) had this effect. Similarly, beads coated with PD184352 suppressed *Krox20 *expression in r5 (Fig. 6h, n = 6/6), while expression remained unaffected after treating with DMSO (Fig. 6f, n = 7/7) or LY294002 (Fig. 6i, n = 6/6). These results indicate that FGF signaling mediates *MafB *and *Krox20 *expression through the Ras-ERK1/2 pathway with no involvement of the PI3K-Akt pathway. As previously shown with SU5402 in solution [1], beads coated with SU5402 abolished *MafB *(Fig. 6b, n = 6/6) and *Krox20 *(Fig. 6g, n = 9/9) in the hindbrain region that was posed in the vicinity to the bead implantation. Importantly, these effects were only observed when embryos were treated with SU5402 before the onset of these genes, 5ss for *MafB *and 6ss for *Krox20 *in r5. Disruption of FGF signaling had no effect on *MafB *or *Krox20 *expression after this time window (Fig. 6e, j, n = 6/6 and n = 5/5 respectively), although it still inhibited *MKP3 *expression (Fig. 5; data not shown). These results suggest that FGF signaling is involved in the induction of *MafB *and *Krox20 *in the caudal hindbrain, but not in their maintenance. As expected from our previous results showing that *Fgf3 *is not dependent on FGF signaling [1], neither SU5402 (Fig. 6l, n = 5/5) nor PD184352 (Fig. 6m, n = 5/5) were able to inhibit *Fgf3 *expression within the hindbrain at these early stages.

## Discussion

Previous results showed that ectopic *vHnf1 *induces expansion of the *Fgf3 *expression domain within the hindbrain, and activates *Krox20 *and *MafB *expression [1]. The current work demonstrates that upregulation of *Fgf3 *upon *vHnf1 *overexpression is a rapid event suggesting that *vHnf1 *directly induces *Fgf3 *transcription. We also analyzed the expression profile of the readouts of FGF activity in the caudal hindbrain and show that they are induced by *vHnf1 *overexpression, confirming the role of *vHnf1 *upstream FGF signaling. In addition, we have demonstrated that FGF signaling in the hindbrain operates through the Ras-ERK1/2 pathway activating *Krox20 *and *MafB *, and FGF3 is the triggering factor.

### Regulation of Fgf3 expression within the hindbrain

We have demonstrated that *Fgf3 *is rapidly induced upon *vHnf1 *overexpression in the hindbrain. These results led us to propose that *Fgf3 *induction is directly regulated by *vHnf1 *in the caudal hindbrain. However, several other players may be involved in regulating the expression of *Fgf3*. This can be inferred from the complex and dynamic expression profile that this gene displays within the hindbrain. Indeed, we previously demonstrated that *vHnf1 *can only induce *Fgf3 *in a discrete period of time that comprises the earliest steps of neurulation, from 0-1ss to 7ss [1]. How *Fgf3 *expression is later controlled remains largely unknown. Mutant mice for *Kreisler *and *Hoxa1 *exhibit reduction in the levels of *Fgf3 *expression in r5 and r6, relating these genes to the regulation of *Fgf3 *[47-49]. In addition to this, a recent report proposes that *Fgf3 *expression in the chick hindbrain requires inhibition of BMP signaling by follistatin and active FGF signaling [50]. However, in our hands, neither electroporation of *mFgf3 *nor loss-of-function of FGF by chemical inhibitors was able to induce or abolish *cFgf3 *at short periods. Most probably, this discrepancy is due to differences in the length of treatment and the embryonic stage of the specimens.

The fact that the *Fgf3 *expression profile within the hindbrain is not exactly coincident in the different vertebrate species [26,47,51,52] suggests that its regulation presents substantial species-specific differences. Interestingly, whereas we have demonstrated that in chick *vHnf1 *overexpression rapidly induces *cFgf3*, the *vhnf1 *hypomorphic mutant in zebrafish shows caudal expansion of *fgf3 *[12]. Detailed characterization of the *Fgf3 *regulatory regions in different species would help to clarify this issue. To the date, trials in this direction have failed to clearly localize the regulatory region/s responsible for *Fgf3 *expression in the hindbrain [25]. We have scanned the annotated chick *Fgf3 *locus to identify potential vHNF1-binding sites in the *Fgf3 *regulatory regions, but DNA sequence analysis did not led us to identify any regulatory region of relevance. BLAST analysis similarly failed to find conserved sequence elements present amongst disparate genomes in the databases. Therefore, identifying the potential vHNF1-binding sites in this region represents a major goal in understanding the molecular mechanisms required for *Fgf3 *expression in the hindbrain.

### A local gradient of ERK1/2 activity coincides with early expression of Fgf3 and FGF activity readouts in the caudal hindbrain

We studied FGF signaling during hindbrain patterning by analyzing the profile of the FGF-activated Ras-ERK1/2 pathway. We show that the activated form of ERK1/2, pERK1/2, is localized at early stages of development in embryonic territories that are within or close to FGF sources, such as: the forebrain influenced by FGF8/12/13 from the ANR [53], the midbrain and the hindbrain influenced by FGF8 and FGF3 respectively, the precardiac mesoderm influenced by FGF8 from the endoderm [54] and the otic placode under the influence of FGF3 from the hindbrain and FGF19 from the mesoderm [32]. This is in agreement with other works in mouse [55] and chick [20], in which the localization of pERK1/2 is compared with the expression profile of *FGFRs1-4*, *MKP3 *and the *Pea3 *subfamily of Ets factors during early embryonic development. HH8 embryos display a pERK1/2 gradient extending anteriorly and posteriorly from the caudal hindbrain. This gradient is consistent with the expression of *Fgf3 *in this territory. At later stages, pERK1/2 is localized throughout the hindbrain, being more intense in the MHB. Therefore, our results suggest that the initial activation of pERK1/2 in the caudal hindbrain depends on a local FGF source within this area rather than being the tail of a gradient established in more rostral areas. This idea is supported by our analysis of the FGF-activity readout *MKP3*. *MKP3 *is expressed in two domains within the hindbrain, a more rostral one in the MHB and a more posterior one in the caudal hindbrain. Between these two domains there is a region of the rostral hindbrain that does not express *MKP3*, suggesting that this region has lower levels of ERK1/2 activity. On the contrary, although *Pea3 *is also initiated in two different domains, at later stages it is expressed throughout the hindbrain. This could reflect that *MKP3 *and *Pea3 *transcriptions require different thresholds of ERK1/2 activity to be induced. We have analyzed the expression of *Spry2 *and *FLRT3*, two other genes belonging to the FGF synexpression group. The negative modulator *Spry2 *is highly expressed in the MHB and r1 but shows weak and very transient expression in the hindbrain (data not shown). This expression does not fully coincide with the expressions of *Fgf3 *or *MKP3 *in the caudal hindbrain since it is slightly anterior to them. The positive modulator of FGF activity *FLRT3 *was observed in the anterior part of the neural tube but not in the caudal hindbrain (data not shown). This gene is later restricted to the MHB [56]. Therefore, genes from the FGF synexpression group are expressed in a dynamic and tissue-dependent manner. Since the role of these genes is to regulate FGF signaling (*MKP3*, *Spry2, FLRT3*) or to modulate gene expression in response to FGF signals (*Pea3*, *Erm *, *Er81*), it is likely that regional and temporal variation in the levels of expression of these genes during embryogenesis can tune FGF signaling in each particular event. How this context-specific tuning is achieved is largely unknown.

### FGF activity in the caudal hindbrain is mediated by the Ras-ERK1/2 pathway with no involvement of the PI3K- Akt pathway

The Ras-ERK1/2 pathway is the most widely reported pathway in FGF-required developmental processes [16,18,27,37,38,57]. In certain cases PI3K-Akt pathway is proposed to act together with the Ras-ERK1/2 pathway in either synergistic or antagonistic manners [17,36,39]. We show that activated effectors of both pathways are present in protein extracts from hindbrains of HH8-9 embryos, suggesting that both pathways are active in this tissue. However loss-of-function studies of these pathways showed that only the Ras-ERK1/2 pathway is involved in the hindbrain patterning.

MKP3 has a very specific function in dephosphorylating ERK1/2 and thus inactivating the Ras-ERK1/2 pathway. Two models, one in the limb development and the other in the isthmic organizer, propose *MKP3 *as a pivotal molecule in mediating crosstalk between PI3K-Akt and Ras-ERK1/2 pathways [17,36]. Both models propose that *MKP3 *is induced by the PI3K-Akt pathway to turn off the Ras-ERK1/2. Our observations demonstrate that in the hindbrain the expression of the *MKP3 *is dependent on the Ras-ERK1/2 pathway but independent of the PI3K-Akt pathway. Thus, in the caudal hindbrain, *MKP3 *would be mainly involved in the autoregulation of the Ras-ERK1/2 pathway rather than in mediating crosstalk with the PI3K-Akt pathway. This is in agreement with a model proposed both in limb development and neural induction [27,28]. Recent work by Ekerot *et al *. strongly supports the hypothesis that *MKP3 *is the mediator of an autoregulatory loop within the Ras-ERK1/2 pathway [58]. This analysis shows that the activation of the *MKP3 *promoter by FGF signaling is ERK1/2-dependent and requires an intact Ets-binding.

*MKP3 *induction is detected as soon as 3 hours after *Fgf3 *overexpression and by 6 hours is expressed throughout the hindbrain. *MKP3 *is also induced 8 hours after *vHnf1 *overexpression suggesting that this gene is regulated by *vHnf1 *through FGF signaling. Conversely, *MKP3 *was downregulated in the caudal hindbrain as soon as 2 hours after blocking FGF signaling or the Ras-ERK1/2 pathway. These timings are consistent with experiments in which FGF-beads grafted in the chick epiblast induced *MKP3 *within 1 and 4 hours [27]. This induction was counteracted within 2 and 4 hours by adding a bead coated with the FGFR inhibitor SU5402 or the ERK1/2 inhibitor PD184352 [28]. Therefore, this indicates that *MKP3 *is a highly sensitive FGF readout that responds quickly to variations in FGF signaling within the hindbrain. This quick response could be important to differentiate between punctual and sustained stimulation of the FGF-ERK1/2 pathway and, therefore, for modulating the different array of responses that this pathway can promote.

**Figure 7:**
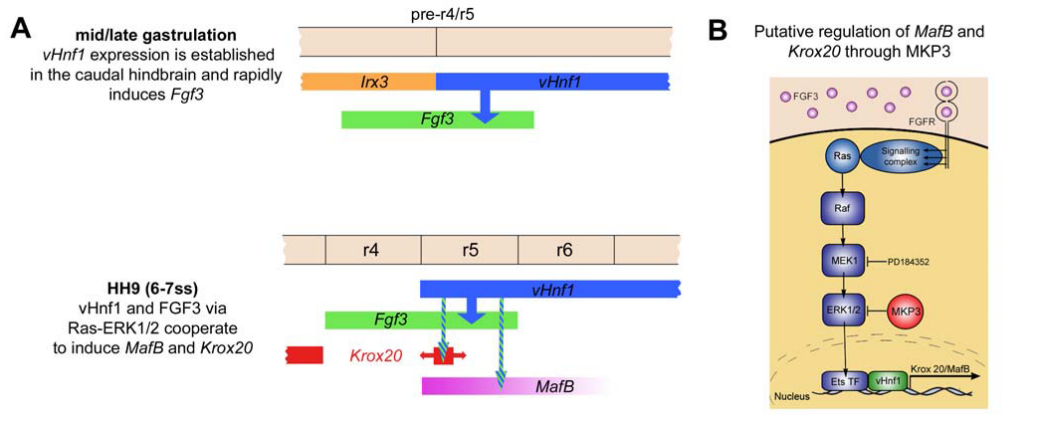
**Model depicting the cooperation of *vHnf1 *and FGF signals in the induction of *MafB *and *Krox20 *in the caudal hindbrain**. (a) *vHnf1 *is very early expressed in the caudal neural plate with a sharp boundary laying in the prospective r4/r5 boundary. This expression may be initiated very early in response to RA from the axial and paraxial mesoderm during mid/late stages of gastrulation [12,64,65]. Anterior limit of expression of *vHnf1 *may be established by mutual repression with an *irx *gene [61,65], probably *irx3 *(unpublished results).*vHnf1 *rapidly induces *Fgf3 *in the caudal hindbrain [1] , which activates the Ras-ERK1/2 pathway. *vHnf1 *and Fgf3-ERK1/2 co-operate for the induction of *MafB *expression in r5 and r6 at 4-5ss and *Krox20 *in r5 at 6-7ss. *Krox20 *induction is probably also dependent on *MafB *expression as suggested in mouse and zebrafish [8,11,66,67]. *Krox20 *is initially expressed in a narrow domain caudal to r4 and subsequently expands its expression area due to non-cell autonomous induction. Coinciding with the onset of *Krox20 *in r5, *vHnf1 *progressively regresses to r6 between 7 and 10ss. Mutual repression between *vHnf1 *and *Krox20 *may prevent expansion of *Krox20 *to r6. Anterior is to the left. (b) Putative regulation of *MafB *and *Krox20 *expression by *vHnf1 *modulating FGF signaling through *MKP3*.

The expression of the rhombomeric markers *Krox20 *and *MafB *is only dependent on the Ras-ERK1/2 pathway. Thus, FGF signaling mediates caudal hindbrain patterning through the Ras-ERK1/2 pathway. Previous findings in zebrafish support this hypothesis: when *vhnf1 *is co-expressed with a constitutively active form of ERK in the zebrafish embryo the *val/MafB *gene is ectopically induced, just as occurs when co-expression of *vhnf1 *and *fgf3 *is performed [12]. In addition, FGF blocking inhibits *Krox20 *and *MafB *expression only if inhibitor treatment is done before the onset of these genes, this is 5ss for *MafB *and 7ss for *Krox20*. Thus, it seems that *vHnf1 *and FGF-ERK1/2 signaling are needed for early establishment of *Krox20 *and *MafB *expression rather that for their maintenance. Consistently, certain evidences suggest that maintenance of *Krox20 *and *MafB *expression depends on autoregulatory mechanisms [14,59,60]. Fig. 7 depicts data obtained in several groups: the first hindbrain molecular boundary is established in r4/r5 by mutual repression of *Irx *and *vHnf1 *[61]; once *vHnf1 *is expressed in the caudal hindbrain, it rapidly induces *Fgf3 *[1]. Finally, *vHnf1 *and *Fgf3 *would cooperate via Ras-ERK1/2 to induce *MafB *and *Krox20 *in the caudal hindbrain [12] and FGF signaling would be regulated by *MKP3*.

## Conclusion

In this report we show that MKP3 is the readout of FGF3 activity in the hindbrain, and Ras-ERK1/2 activity is necessary for *MKP3, Krox20 and MafB *induction. Therefore, these results suggest that the Ras-ERK1/2 pathway is involved in mediating FGF signaling during caudal hindbrain patterning with no apparent contribution of the PI3K-Akt pathway. Taken together, the data presented in this work and previous knowledge leads us to propose a model in which *vHnf1 *is the molecular switch that initiates the process of r5 and r6 specification. *vHnf1 *rapidly induces *Fgf3 *in the caudal hindbrain, which activates the Ras-ERK1/2 pathway. *vHnf1 *and *Fgf3-ERK1/2 *co-operate for the induction of *MafB *expression in r5 and r6 at 4-5ss and *Krox20 *in r5 at 6-7ss.

## Methods

### Embryos and staging

Chick embryos were obtained from fertilized hens' eggs (Granja Gibert, Tarragona, Spain) and incubated in humidified atmosphere at 38°C. Embryos were staged according to Hamburger and Hamilton [62]. All procedures used have been approved by the institutional animal care and use ethic committee (PRBB-IACUC), and implemented according to national rules and European regulations.

### In ovo electroporation

*mvHnf1-IRES-GFP *, pCS2-*mFgf3 *and pCAβ-*EGFP *expression constructs, were overexpressed into the hindbrain of HH7-9 chick embryos by *in ovo *electroporation. A solution containing the construct (2 μg/μl) was mixed 1:1 with Fast Green (1 μg/μl). By using a micropipette (GC150-15 capillaries, Clark electromedical instruments, pulled with a Narishige Japan puller), the plasmid solution was seeded on the top of the neural plate or microinjected in the lumen of the neural tube. A platinum cathode was placed at the left side while the anode was placed at the right side of the embryo. 4 square pulses (5, 10 or 20V) were generated by an electroporator Square CUY-21 (BTX Co., Ltd, Tokiwasaiensu, Japan). M199 medium (Gibco) was added immediately after electroporation to protect the embryo from dryness. Eggs were incubated in humidified atmosphere at 38°C the desired time period. After that, embryos were collected in cold Phosphate Buffered Saline (PBS pH7.4), selected for GFP fluorescence under the microscope, and treated for RNA extraction or fixed overnight in 4%paraformaldehyde (PFA) in PBS for further analysis.

### RNA extraction from fresh tissue

Electroporated chick hindbrains and mouse hepatic tissue were isolated and placed in Trizol (Invitrogen). RNA was isolated from Trizol using the chloroform extraction protocol provided by Invitrogen.

### Semiquantitative RT-PCR

One-step PCR kit (Qiagen) was used to amplify specific sequences of *mvHnf1*, *cFgf3 *and *cGAPDH *. Primer sequences were: 5' AGAGCTGCCCTGTACACTTG, 5' CATGGTGACTGATTGTCGAA for *mvHnf1*;

5' CCTTGGAGAAAAACAGCGTC and 5' AGCGTCCTCTCCTTCTCCTC for *cFgf3*; 5' TACTGGAATGGCTTTCCGTGT and 5' ACTTTATTGATGTAAGGTGGTACAC for *cGAPDH *. The RT-PCR program was the following: 30 min 50°C; 15 min 95°C; 1 min 94°C 1 min 58°C 1 min 72°C for 22, 25, 27 or 30 cycles; 10 min 72°C. 100 ng of total RNA was used per sample. In each case, *mvHnf1 *and *cFgf3 *were amplified in the same tube. A series of experiments were performed to narrow down the number of amplification cycles needed to visualize the genes of interest. Amplification products were run in a 1.5% agarose gel. *cGAPDH *amplification was used to normalize samples. The amplified bands were quantified using the Quantity-one software (Biorad) and the percentage of volume (%V) of each band was obtained according to: [band V/total V] × 100. The volume of the band was measured as the sum of the intensities of all the pixels in a given area. Before calculating the %V, the volumes were adjusted by subtracting the part corresponding to the background. The relative level of *cFgf3 *expression was estimated by calculating the ratio between %V of the *cFgf3 *band and %V of the *mvHnf1 *band in each sample.

### Treatment of Jurkat cells or whole embryos with specific inhibitors

Jurkat cells or whole embryos were used to assay the activity of FGF-signaling inhibitors. Cells were stimulated via their TCR/CD3 complex to highly activate both the Ras-MAPK and the PI3K FGF-dependent pathways [42-44]. 10^5 ^cells per sample were incubated during 1 h with DMSO, 25 μMSU5402 (Calbiochem), 40 μM LY294002 (Calbiochem) or 20 μM PD184352 (University of Dundee) and processed for western blot.

### Whole embryo organotypic explants and bead implantation

Collagen beds were prepared in 4-well dishes (Nunclon) by adding a drop of 10 μl of Matrigel preparation (Invitrogen) in the center of each well. HH7-8 embryos were collected and dissected in M199 medium. Organotypic explants were prepared by cutting an area around the embryo that comprised the entire *area pellucida *and part of the *area opaca *. This kind of dissection ensures a good survival and development of the embryo and makes it easy to attach to the Matrigel support. Explants were transferred to the 4-well plates and positioned with the dorsal side to the top and M199 was substituted by DMEM medium. AGI-X2 formate beads (Bio-Rad) were coated with the following inhibitors: 5 mM SU5402, 20 mM LY294002 or 10 mM PD184352 [28] for 1-2 h at RT, protected from light and washed in PBS before grafting. DMSO beads were used as controls. Beads were grafted next to the hindbrain region of the explanted embryos by using thin forceps. Explants were incubated at 37.5°C in a water-saturated atmosphere containing 5% CO_2 _during 2 h, 4 h, 6 h or 8 h. After that, explants were processed for western blot or fixed overnight in 4%PFA/PBS for further analysis.

### Whole mount in situ hybridization

Whole mount in situ hybridization was carried out using digoxigenin-labeled riboprobes as previously described [63]. Digoxigenin was detected with NBT/BCIP (Roche), which provides a purple stain. Riboprobes were as follows: *cFgf3 *(dEST Data Bank), *cKrox20 *[59], *cMafB *[60], *cMKP3 *[36], *cPea3 *[18] and *cSpry2 *[34].

### Immunohistochemical detection of Green Fluorescent Protein (GFP) or phosphorilated ERK1/2

Embryos were collected in cold PBS and rapidly fixed with 4%PFA/PBS overnight at 4°C. They were washed with PBS, dehydrated to 100% methanol, stored during 1 h at -20°C and rehydrated to PBS. Afterwards, they were treated with 6% H_2_O_2_/PBTx (PBS 1%triton) 2 h at rt, washed in PBTx, incubated in blocking solution (PBTx 10% heat inactivated goat serum) for 1 h and subsequently incubated either with polyclonal antibody anti-GFP [1:500] (Molecular Probes) overnight at 4°C [34], or anti-dual phosphorylated (dp)ERK1/2 [1:50] (Cell Signaling) during 5 days at 4°C. Donkey anti-rabbit conjugated with horseradish peroxidase [1:200] (Amersham) was used as secondary antibody for α-GFP experiments. In anti-pERK stainings, biotinylated [1:50] (Vector) or Alexa488 anti-rabbit antibodies (Molecular Probes) were used. Embryos were subsequently washed with blocking solution and incubated with the ABC kit (Vector) overnight at 4°C. To develop the peroxidase activity a colorimetric reaction was carried out with the AEC substrate system (Lab Vision). Controls with no primary antibody were performed in parallel to the experiment.

### Western blots

Jurkat T cells (10^5^cells/sample) or hindbrain tissue from HH7^+^-HH9 chick embryos (pools of 10 embryos/sample) were dissociated in 2 mM EDTA/PBS, homogenized in 10%SDS/Sample buffer and kept at -80°C. After 5 min at 100°C the lysates were run in a 12.5% polyacrilamide gel and transferred to Immobilon-P PVDF membrane (Millipore). Membranes were incubated with antibodies to phosphorilated ERK1/2 [1:1000] (Cell Signaling), total ERK1/2 [1:5000] (Promega), phosphorilated Akt [1:5000] (Cell Signaling) and total Akt [1:5000] (Cell Signaling). The secondary antibody was donkey anti-rabbit conjugated with HRP [1:2000] (Amersham). Chemiluminescence developing reagents were West Pico or West Femto (Pierce Laboratories).

### Photography and imaging

Whole, flat-mounted or sectioned embryos were photographed using Leica DMR fluorescence microscope or Leica, MZ FL III fluorescence scope both with Leica DFC 300FX cameras. Images were captured with *Leica IM50 v4.0 *and analyzed with *Adobe Photoshop v7.0.1*.

## Authors' contributions

FA carried out the experimental studies and participated in the design of the study. CP conceived the study, participated in its design and coordination and drafted the manuscript. All authors read and approved the final manuscript.
